# SOCIAL ISOLATION, LONELINESS, AND DEPRESSIVE SYMPTOMS AMONG OLDER ADULTS: THE MODERATING EFFECT OF RESILIENCE

**DOI:** 10.1093/geroni/igae098.0288

**Published:** 2024-12-31

**Authors:** Ke Li, Fengyan Tang, Steven Albert, Mary Rauktis, Mary Ohmer

**Affiliations:** University of New Hampshire, Durham, New Hampshire, United States; University of Pittsburgh, Wexford, Pennsylvania, United States; University of Pittsburgh, Pittsburgh, Pennsylvania, United States; University of Pittsburgh, Pittsburgh, Pennsylvania, United States; University of Pittsburgh, Pittsburgh, Pennsylvania, United States

## Abstract

Social isolation has been recognized as a social problem with negative health consequences. Using data from three waves of the Health and Retirement Study, this study aimed to examine the long-term impact of social isolation on loneliness and depressive symptoms and to explore the moderating effect of resilience. This study comprised 3,681 U.S. adults aged 60 and older at the baseline wave. Social isolation index was constructed using five indicators, including not married or cohabitating with a partner, no social participation, and less than monthly contacts with children, family members, or friends. Loneliness was measured by the UCLA loneliness scale and depressive symptoms were measured by the Center for Epidemiological Studies-Depression scale (CES-D). The moderator of resilience was assessed by the simplified resilience score (SRS). Latent growth curve models with robust standard errors were estimated. The results of latent growth curve models showed that social isolation was significantly associated with more initial loneliness and depressive symptoms. However, social isolation was associated with a slower increasing rate of loneliness, but no significant relationship with the change rate of depressive symptoms. Furthermore, resilience significantly buffered the negative effect of social isolation on the initial level of depressive symptoms. The findings underscore the importance of enacting strategies and interventions targeting resilience to address social isolation and its negative consequences among older adults.

